# Revisiting the evidence on CT-guided biopsy and percutaneous endoscopic debridement for spondylodiscitis

**DOI:** 10.1016/j.bas.2025.104182

**Published:** 2025-01-08

**Authors:** Aaron Lawson McLean

**Affiliations:** Department of Neurosurgery, Jena University Hospital – Friedrich Schiller University Jena, Jena, Germany

Dear Editor,

I read with great interest the recent systematic review by Acharya et al., which compares CT-guided needle biopsy (CTNB) and percutaneous endoscopic debridement and drainage (PEDD) for managing spondylodiscitis (SD) (Acharya et al., 2024). While this study tackles an important clinical question, several methodological concerns deserve attention to better clarify and strengthen the reliability of its findings.

One major issue lies in the inclusion of multiple studies from the same research groups, notably those led by Yang et al. and Fu et al. These studies, conducted at the same institution over overlapping periods, lack clear assurances that they involve distinct patient cohorts. Without such confirmation, there is a significant risk of patient overlap, which could artificially inflate sample sizes and undermine the statistical independence crucial for meta-analysis.

Another concern is the marked heterogeneity between the patient groups analyzed. The PEDD cohort primarily consists of patients with lumbar infections who present predominantly with localized back pain, while the CTNB cohort spans infections across the cervical, thoracic, and lumbar spine, often accompanied by systemic symptoms such as fever or sepsis. These baseline differences are likely to confound the study outcomes, as factors like infection location and severity significantly influence pathogen detection rates and clinical improvement. Without subgroup analyses or statistical adjustments for these variables, the conclusions may reflect these biases rather than true differences between the interventions.

Finally, the review's methodological rigor appears insufficient. The authors did not employ established quality assessment tools, such as the Newcastle-Ottawa Scale or Cochrane Risk of Bias tool, leaving the validity of the included studies unclear. Furthermore, essential statistical evaluations, such as heterogeneity assessments (e.g., I^2^ statistics) and a justified choice of random- or fixed-effects models, are missing, making the pooling of data difficult to evaluate.

In conclusion, while Acharya et al. address an important clinical question and propose PEDD as a viable alternative to CTNB, particularly for pathogen identification and pain relief, the methodological limitations and potential biases in their review constrain the reliability of their conclusions. Until more robust, high-quality evidence becomes available, clinicians should interpret these findings cautiously and continue to base decisions on established guidelines and individualized patient assessments, given the procedural differences and varying clinical presentations inherent to this condition.

## Declaration of competing interest

The authors declare that they have no known competing financial interests or personal relationships that could have appeared to influence the work reported in this paper.
